# Aiding Cancer’s “Sweet Tooth”: Role of Hexokinases in Metabolic Reprogramming

**DOI:** 10.3390/life13040946

**Published:** 2023-04-04

**Authors:** Zeenat Farooq, Hagar Ismail, Sheraz Ahmad Bhat, Brian T. Layden, Md. Wasim Khan

**Affiliations:** 1 Division of Endocrinology, Diabetes, and Metabolism, Department of Medicine, The University of Illinois at Chicago, Chicago, IL 60612, USA; 2 Jesse Brown Veterans Affairs Medical Center, Chicago, IL 60612, USA

**Keywords:** cancer metabolism, HKDC1, hexokinases, glucose metabolism, metabolic reprogramming

## Abstract

Hexokinases (HKs) convert hexose sugars to hexose-6-phosphate, thus trapping them inside cells to meet the synthetic and energetic demands. HKs participate in various standard and altered physiological processes, including cancer, primarily through the reprogramming of cellular metabolism. Four canonical HKs have been identified with different expression patterns across tissues. HKs 1–3 play a role in glucose utilization, whereas HK 4 (glucokinase, GCK) also acts as a glucose sensor. Recently, a novel fifth HK, hexokinase domain containing 1 (HKDC1), has been identified, which plays a role in whole-body glucose utilization and insulin sensitivity. Beyond the metabolic functions, HKDC1 is differentially expressed in many forms of human cancer. This review focuses on the role of HKs, particularly HKDC1, in metabolic reprogramming and cancer progression.

## 1. Introduction

First observed by Otto Warburg in 1924, one of the hallmarks of cancer cells is reprogrammed glucose metabolism, where glucose uptake and lactate production are enhanced regardless of oxygen concentrations, popularly known as the “Warburg effect.” Initially, this phenomenon was thought to be due to mitochondrial dysfunction in cancer cells [1]. However, research has now established that enhanced glucose metabolism coupled with altered mitochondrial metabolism supplies increased energy needs and provides metabolites for biosynthetic pathways, such as nucleotides, fatty acids, and amino acids needed by proliferating cancer cells [2]. The first step of glucose metabolism is catalyzed by hexokinases (HKs). HKs are a family of phosphotransferase enzymes with different kinetic properties, expression profiles, and subcellular localization that initiate glucose metabolism [3,4,5]. Four canonical isoforms of the HK family have been well characterized: HKs 1–3 have a broad range of expression, and the fourth isoform, more commonly known as glucokinase (GCK), is expressed mainly in the liver and pancreas [3,4,5,6]. Although specific roles have been described for each HK, the existence of multiple isozymes catalyzing the same reaction within the same cell or tissue is a pressing question. Glucose-6-phosphate (G6P) is the first stable intracellular intermediate of glucose metabolism; therefore, its generation is tightly regulated by the selective expression of different HK isoforms in normal and pathophysiological scenarios [7]. For the same reason, HKs vary in cellular distribution, expression patterns, and substrate affinity levels depending on the cell’s physiological state. This review describes the role, distribution, and regulation of different isoforms and their metabolic functions in cancer.

### 1.1. General Characteristics and Distribution

**Figure 1:**
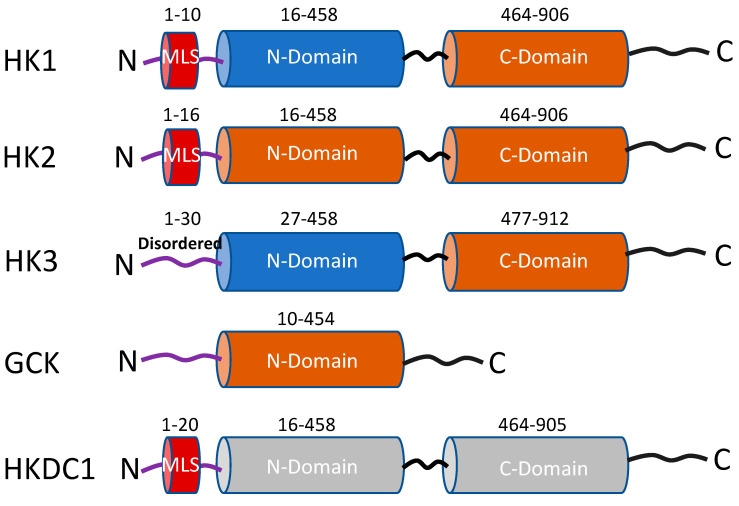
**Schematic representation of the functional domains of the five hexokinase isoforms.** The rust-colored cylinders represent domains with catalytic activity, and the blue cylinders have no catalytic activity. Both cylinders in HKDC1 are gray colored because this isoform has very low kinase activity. MLS = mitochondrial localization sequence (red-colored cylinder). Numbers represent amino acid sequences adapted from Uniprot.org.

Genes that code for HK protein isoforms are conserved from bacteria to humans [3,5]. However, bacterial and lower vertebrate genes code for smaller proteins (about 50 kDa), whereas mammalian HKs 1-3 and HKDC1 are ≅100 kDa in size. HK isoforms possess high sequence similarities (Supplementary Figure S1) at the ‘N’ and ‘C’ terminal domains referred to as “Hemi domains” (Figure 1) [3,4,5,8,9,10,11,12,13,14,15,16,17,18,19,20,21]. These hemi-domains are thought to have evolved because of a gene duplication event from the bacterial HK enzyme [22,23]. Upon subsequent evolutionary divergence, the N-terminal hemi-domain acquired different properties in each isoform (Figure 1) [8,9,10,11,12,13,14,15,16,17,18,19,20,21,24,25]. One characteristic feature of mammalian HKs is allosteric inhibition by G6P, which supports the “gene duplication event theory,” suggesting that the duplication event led to the formation of a hemi-domain that evolved into a regulatory binding site [26]. Amino acid sequence comparisons of hexokinases from lower to higher vertebrates are available to support the gene duplication hypothesis (Supplementary Figure S1). Various comparison analysis studies suggest that high similarities in sequence exist not only between the hemi-domains but HKs from lower to higher vertebrates, indicating a slow rate of amino acid substitution (rate of mutation through evolution) at homologous HK genes across species [26,27,28]. Some of the common characteristics of each isozyme are described and listed in Table 1. The HK1 gene encodes a protein of 100 kDa, and only the C-terminal domain is catalytically active [3,5]. It is ubiquitously expressed inside all cells with a granular cytoplasmic expression pattern. The enzyme is also localized to the mitochondrial outer membrane [3,4,5]. HK1 has the highest level of tissue expression in the brain, followed by the urinary bladder, thyroid gland, colon, and bone marrow [3,5]. HK2 is the most well-characterized isoform of the HK family, primarily expressed in insulin-sensitive tissues such as adipose and skeletal muscle [29]. It undergoes significant changes in expression in different cancers and is the most well-studied HK in cancer biology [30,31,32,33,34,35,36,37,38,39,40]. It is the only identified HK with both N and C terminal domains catalytically active and is the most highly regulated isoform [3,4,5,9,10,11]. Like HK1, HK2 has been shown to localize to mitochondria [5]. HK3 is a less well-characterized 100 kDa isoform of the hexokinase family, which lacks the N-terminal mitochondrial binding domain of HK1 and 2 (Figure 1). HK3 is expressed in lung, kidney, and liver tissues at lower levels than HK1 and 2. It is also the predominant isozyme in granulocytes [10,11,41] (Table 1). HK4, or glucokinase (GCK), is a unique 50 kDa enzyme mainly expressed in the liver and pancreas and closely resembling the ancestral bacterial enzyme [42,43]. The enzyme is also expressed in enteroendocrine cells and the brain [44,45]. The distinguishing feature of GCK in metabolic regulation is its role as the body’s primary glucose sensor. Small fluctuations in GCK activity alter the threshold for glucose-stimulated insulin secretion (GSIS) from pancreatic β-cells, which is not observed with other hexokinases [11,28,46]. Mutations in the GCK gene lead to two different diseases of blood glucose regulation: maturity-onset diabetes of the young type 2 (MODY-2), and persistent hyperinsulinemic hypoglycemia of infancy (PHHI) [47,48,49]. GCK is localized in the cytoplasm, but reports have also suggested that GCK forms a heteropentameric complex at the mitochondria with BCL 2-associated death promoter (BAD), protein kinase A (PKA, cAMP-dependent protein kinase), protein phosphatase 1 (PP1, dual-specificity serine/threonine phosphatase), and Wiskott–Aldrich family member (WAVE1) under certain conditions to integrate glycolysis and apoptosis [50,51].

Phylogenetic analyses carried out in the middle of the 2000s to comprehend the diversification of the HKs and the evolution of GCK [19,20] led to the discovery of a novel HK gene known as the hexokinase domain containing-1 (HKDC1). The gene that codes for HKDC1 lies on chromosome 10 in humans near the HK1 gene, and the two share more than 70% sequence similarity [20]. The enzyme has been shown to play a role in modulating glucose tolerance during pregnancy by identifying its genetic variants in a genome-wide association study (GWAS) [13,18]. Like HK1-2, it also contains an ‘N’ and a ‘C’ terminal domain, which are the regions predicted to bind glucose and ATP, respectively. It includes amino acid residues, which remain conserved with those of the other HKs (Figure 1). [19,20]. HKDC1 is broadly expressed in the retina of the eyes, kidneys, brain, small intestine, duodenum, pharynx, esophagus, and thyroid gland [20]. We [14,16,52] and others [53] have shown that HKDC1, like HK1-2, associates with the outer mitochondria membrane via its interaction with the voltage-dependent anion channel (VDAC). 

### 1.2. Regulation of Hexokinase Expression

The primary metabolic role of HKs is the phosphorylation of glucose, thus trapping it inside cells and initiating glucose metabolism [3,4,5,54]. HKs, therefore, dictate the direction of glucose flux within the cells. However, if the phosphorylation of glucose were the only role of HKs, the presence of a single HK would seem reasonable. The existence of different isoforms raises a question about the non-redundant role played by each enzyme. It also suggests that different rates of glucose phosphorylation are needed depending on the cell/tissue requirements. This allows “metabolic plasticity” to the cells to allow better regulation and channeling of glucose metabolism, and the role of GCK in this context has been defined most elaborately [6]. Therefore, the expression of HKs is profoundly altered in cancer cells, and it varies widely among different cancer types (Supplementary Figure S2).

Transcriptional Control: This can be illustrated by highlighting differences in transcriptional regulation between different isoforms. Promoter regions of different hexokinases have been analyzed to contain several regulatory elements governing different transcription profiles under varied conditions [55]. The isoforms have also been observed to bind multiple transcriptional factors [56,57,58,59,60,61,62,63]. The promoter of HK1 in rats has been shown to contain transcriptional start site elements, lack a TATA sequence, and lie within a CpG island that extends into the translational start site [64]. These features are similar to promoter element features of housekeeping genes, tailored for their ubiquitous expression [64]. HK1 promoter also contains regulatory sites known as sp sites within the P2 BOX, which are essential for promoter activity and the binding of protein factors in lower vertebrates to humans [57,65,66]. The role of non-coding elements in regulating HK1 and their association with congenital hyperinsulinism has also been reported [65]. Alternative splicing generates multiple HK1 isoforms, i.e., HK1 (ubiquitous), HKR (erythrocytes), HK-TA/TB (testis-A/B), HK-TB (testis-B), and HK-TD (testis-D). HK1 and HKR isoforms differ only in exon 1 and share the remaining 17 exons, whereas HK-TA, HK-TB, HK-TC, and HK-TD have different 5′ UTR exons share the 17 exons with all other isoforms of HK1 [64]. On the other hand, the promoter region of HK2 contains a single transcriptional start insulin-binding element, leading to the transcriptional upregulation of HK2 by insulin [56]. However, the promoter of HK2 contains a binding motif for hypoxia-inducible factor 1α (HIF1α). HIF1α is upregulated due to hypoxia brought about by the tumor microenvironment, which results in the upregulation of HK2, making HK2 the most highly expressed HK in multiple tumors. Recently, it has been reported that HIF1α is negatively regulated by the long non-coding RNA LINC00365 in breast cancer cell lines, resulting in a decline in HK2 levels and cellular proliferation [67]. The enhanced expression of HK2 in hepatocellular carcinoma in rat models has also been reported to be induced by a loss of DNA methylation on the CpG island on the HK2 promoter [68]. The post-transcriptional regulation of HK2 activity by MicroRNAs has also been documented. Anti-tumorigenic microRNA miR 143 acts on HK2 mRNA, leading to its degradation and decreased stability. The upregulation of oncogenic microRNA miR155 occurs in multiple tumors, repressing miR 143, thereby stabilizing HK2 mRNA [69,70]. HK3 contains a binding site for the basic leucine zipper transcription factor CCAAT/enhancer binding protein alpha (CEBPA) which leads to its transcriptional upregulation during all-trans retinoic acid (ATRA)-mediated neutrophil differentiation [41]. Interestingly, the HKDC1 promoter contains high levels of epigenetic marks such as H3K4me1 and H3K27ac in multiple human cell lines. The regulatory roles of these marks on their expression in normal and cancer cells need further exploration [21]. 

Most cancers shift their HK expression profiles in favor of HK2, a consequence of metabolic re-programming in cancer. This could be illustrated by the induction of gene expression of HK2 and the silencing of GCK in liver and pancreatic cancers [71,72,73,74,75]. In humans, progression from the normal brain to low-grade gliomas and finally to glioblastoma multiforme (N) occurs with a progressive shift from HK1 to HK2 with a concomitant decrease in prognosis. HK2 expression levels are closely associated with tumor grade and mortality in hepatocellular carcinoma and breast metastasis [30,40]. One of the reasons for this response is the catalytic activity in both domains in HK2, favoring greater utilization of glucose and maintaining a downhill gradient for glucose phosphorylation. Additionally, HK2 expression in cancers is stimulated by the insulin signaling pathway Akt/mTORC1, which is upregulated in tumor cells to regulate glucose metabolism, cellular growth, and survival through the phosphorylation of target molecules. Akt phosphorylates HK2 at Threonine 473, which lies within the Akt consensus-binding motif RARQKT*, stabilizing HK2 protein. This motif is conserved from mice through humans [76]. For the same reason, the expression of HK2 is decreased in type 1 diabetes mellitus (T1DM) due to reduced insulin signaling and is recovered upon insulin treatment in T1DM [56,77,78,79,80]. Although HK1 and HKDC1 share the mitochondrial localization property with HK2, they lack the Akt consensus motif, making them more susceptible to degradation through apoptosis. However, we have previously reported that in mouse models, overexpressing HKDC1 enhances Akt phosphorylation (15,16).

### 1.3. Regulation of Hexokinase Activity

HK isoforms have different catalytic and regulatory properties. HK1 is activated by high inorganic phosphate levels (Pi) and inhibited by the product G6P. Therefore, a cellular milieu with a high ratio of Pi/G6P because of high rates of ATP utilization favors glycolysis through HK1 activity for the generation of ATP [5]. One of the best examples to illustrate this is the reversal of the G6P-induced inhibition of HK1 by inorganic phosphate (Pi), which leads to the evasion of the G6P-induced feedback inhibition of glucose phosphorylation and favors its ubiquitous expression since glycolysis is a primary requirement of all mammalian cells [3,4,5].

On the other hand, HK2 lacks this antagonizing response by Pi, and instead, Pi adds to the inhibition caused by G6P in the case of HK2 [45]. This feature favors HK2 activity in metabolically active tissues such as skeletal muscles to replenish glycogen synthesis following muscle contraction. Existing literature suggests an anabolic role for HK2 [11,45]. Additionally, a wealth of literature agrees with an anabolic role for HK2, funneling G6P to synthesize NADPH for lipid biosynthesis via the pentose phosphate pathway (PPP) in the liver and mammary glands [81,82]. 

HK3 is known to be inhibited by glucose at high concentrations of 1 mmol l^−1^ (substrate inhibition) but is less sensitive to inhibition by G6P. Interestingly, HK3 responds towards G6P and Pi similarly to HK2 (Table 1), which supports an anabolic role for HK3; further research is needed to answer this question [28,45]. It also has the lowest affinity for the second substrate, ATP, among all HKs, but the physiological role of this property remains elusive [45].

GCK has the highest Km (lowest affinity) for glucose among all canonical HKs (HK1-4), allowing the liver and pancreas to serve as a “glucose buffer” and a “glucose sensor,” respectively. It is not inhibited by G6P and has a 50-fold lower affinity for glucose than other isoforms. Within the liver, the low affinity is tailored to ensure the availability of glucose to physiologically sensitive tissues such as the brain under starvation and its utilization only when glucose is abundantly available. Within the pancreas, this feature allows GCK to act as a “*glucose sensor*” to regulate insulin release. Mutations in the glucokinase (GCK) gene lead to maturity-onset diabetes of the young, type 2 (MODY-2), and persistent hyperinsulinemic hypoglycemia of infancy (PHHI) [47,49,83]. MODY-2 is a mild type 2 diabetes resulting from a defect in glucose-induced insulin secretion [47,48,49]. Mutations in the GCK leading to MODY-2 are arguably the most common cause of monogenic diabetes due to these specific mutations. More than 40 mutations have been linked to MODY-2, including frameshifts, nonsense, missense, and splice-site variants [1,2,3,4,5,6,7]. The proposed role of GCK as a “glucose sensor” in pancreatic β-cells [2,11,12] is consistent with the MODY-2 phenotype wherein small reductions in β-cell activity increase the threshold for glucose-induced insulin secretion resulting in the phenotype. However, a report by Postic et al. suggests that hepatic GCK also plays a role in MODY-2. Alterations in GCK activity are also associated with many other diseases that have been reviewed elsewhere in detail [12,42]. Owing to its unique role, GCK regulation is complex, and several regulatory mechanisms have been discovered. Alternative and tissue-specific promoters drive GCK transcription and gene expression to varying degrees [84,85,86,87,88,89]. Several metabolites, including insulin, glucose, and hormones, regulate GCK expression at the transcriptional level [43,90,91,92,93]. The regulation of GCK has been recently reviewed elsewhere in more detail [94].

Not much is known about the kinetic and regulatory properties of HKDC1, and it needs further exploration. However, the genetic locus near HKDC1 is a “hot spot” for various “histone modifications,” and it is believed that HKDC1 is subject to different levels of regulation under different physiological and pathophysiological conditions [18,21]. Although HKDC1 has two kinase domains like HK1, there have been contrasting reports on its catalytic potential. An early study suggests that HKDC1 possesses hexokinase activity, where experiments on INS-1 rat pancreatic cells with HKDC1 overexpression showed changes in HK activity [21]. Interestingly, the hexokinase activity of the other HKs was unaffected by the expression of HKDC1 [21]. Going further, our group has recently shown that the hexokinase activity of HKDC1 is quite low, and the principal function of the protein may be more related to binding to mitochondria and modulating glucose flux [16].

### 1.4. Differences in Subcellular Localization

This feature allows the utilization of different HK isoforms for channeling G6P to pathways dictated by the cell’s metabolic state. Under normal conditions, GCK is primarily cytosolic [95], whereas HK3 is mostly perinuclear in localization [96]. The subcellular localization of HK1 and 2 has important influences on their metabolic, antioxidant, and anti-apoptotic effects. HK1 localizes to the mitochondrial membrane, and HK2 is localized to the outer mitochondrial membrane through a voltage-dependent anion channel (VDAC) [3,5]. However, HK2 binds to mitochondria with less affinity than HK1 and can translocate between cytoplasm and mitochondria depending on glucose and glucose 6-phosphate [7]. Mitochondrial-bound HK1 promotes efficient glucose catabolism by coupling glycolysis with oxidative phosphorylation. This feature makes it the ideal HK isoform for brain cells. On the other hand, HK2 in normal cells is mostly cytosolic and promotes anabolic functions such as glycogen synthesis through PPP, making it ideal for muscle and cardiac cells. Additionally, PPP leads to the generation of reduced glutathione from NADPH which is essential for the antioxidant activity of HK2. Although the mitochondrial binding property of HK2 appears to be in tune with the metabolic demands of cancer cells, allowing them to couple glucose consumption with energy (ATP) production, its role in mediating glucose consumption and anabolic processes under normal conditions remains elusive [3,4,5,30,31,32,33,34,35,36]. Studies, however, show that HK2 dynamically shuttles between the mitochondria and cytoplasm in response to changes in intracellular G6P, pH, and Akt signaling pathways [97].

As a result of low-grade inflammation (aging and diabetes), HK1 has been shown to predominantly localize in the cytoplasm and favor an inflammatory phenotype [98,99]. In a landmark study conducted by De Jesus et al., it was observed that mice lacking the N-terminal mitochondrial binding domain (MBD) on HK1 produced an inflammatory response when challenged with lipopolysaccharide (LPS), increased glucose flux through the PPP but decreased flux below the level of glyceraldehyde phosphate dehydrogenase (GAPDH) brought about by the nitrosylation of GAPDH which leads to reduced GAPDH activity [100]. HK3 has also been shown to be associated with mitochondrial-associated membranes (MAMs) in normal mice brains through unknown mechanisms. This effect is abolished due to chronic stress in mice [101]. It has been reported that hexokinases are differentially translocated within cells depending upon the physiological conditions and the mechanisms through which HKs migrate between cellular compartments; however, they remain unidentified and warrant more investigation in this area [102]. Recently, HKDC1 has also been shown to bind with mitochondria via interaction with VDAC [15,16,17,18]. More research is needed in this area to understand better the significance of the differential localization of hexokinases under different conditions.

### 1.5. Roles of Hexokinases in Cancer-Mediated Metabolic Reprogramming

One of the characteristic features of cancer is unabated cell division. For this reason, neoplastic cells preferentially obtain energy and biomolecules through glycolysis through metabolic reprogramming. Metabolic reprogramming refers to the ability of cancer cells to alter their metabolism to support their enhanced metabolic requirements of high ATP and intermediates for biosynthetic processes. This requirement brings about extensive changes in the expression of different hexokinase enzymes.

**Figure 2:**
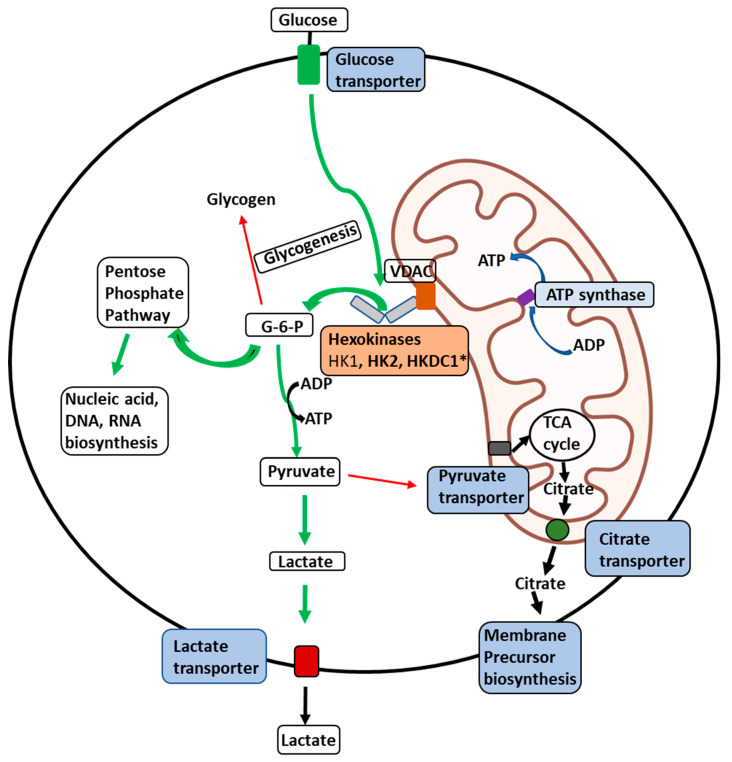
**Illustration of the delivery of glucose to membrane-bound HKs in malignant cells.** Illustration of the glucose delivery to HKs 1, 2, and HKDC1 bound to the outer mitochondrial membrane (OMM) and metabolic fates of the glucose-6-phosphate (G6P) formed thereof within a malignant cell. Glucose transport across the plasma membrane by glucose transporters is phosphorylated by HKs (HK1, HK2, or HKDC1) bound to a voltage-dependent anion channel (VDAC) located on the outer mitochondrial membrane. VDAC allows direct access of ATP generated by the ATP synthase within the mitochondria to the HKs, which can be transported across the inner-mitochondrial membrane by the adenine nucleotide translocator. To maintain malignant cells’ highly glycolytic metabolic flux, the product G6P is rapidly distributed across key metabolic routes (see thick green arrows). The primary metabolic routes for G6P are either entry into the pentose-phosphate pathway for biosynthesis of nucleic acid precursors or conversion to pyruvate and lactate through glycolysis. In cancer cells, most lactate is transported out of the cells with the aid of lactate transporters. In contrast, small amounts of pyruvate are transported to mitochondria through the pyruvate transporters to supply intermediates to the tricarboxylic acid (TCA) cycle (thin red arrows). Citrate transporters transport citrate produced in the TCA cycle to aid in synthesizing membrane components such as phospholipids and cholesterol, essential for tumor cell proliferation. * Novel hexokinase, HKDC1, with roles still unknown.

*Hexokinase 1:* The expression of HK1 is amplified in some cancers where it is responsible for rewiring the metabolic state towards aerobic glycolysis to supply ATP and macromolecules (Figure 2) [103,104,105]. The observation that most normal cells express HK1 while cancer cells express HK1 and HK2 stimulated interest in reducing HK2 activity in cancers. However, studies have demonstrated that the knockdown of HK2 alone does not inhibit in vivo tumor progression with reduced glucose consumption, suggesting that HK1 compensates for the overall tumorigenic potential. In contrast, the knockdown of HK2 in HK1- HK2+ cancers reduced xenograft tumor progression [106,107,108,109]. These studies suggest a greater involvement of HK1 in tumor progression beyond its currently known role and possibly as a regulatory function in cancer cells. For example, in a study by Daniela et al., HK1 has been shown to be involved in ovarian cancer in a glucose phosphorylation-independent fashion [110]. HK1 also serves as the effector of KRAS4A, an isoform of the most frequently mutated oncogene KRAS, during tumorigenesis [111].

*Hexokinase 2:* HK2 is significantly overexpressed in treatment-resistant primary and metastatic breast cancer [37,38,39,40], bladder cancer [112], cervical squamous cell carcinoma [113], colorectal cancer [114], neuroendocrine tumor [103], ovarian epithelial tumors [104], glioblastoma [55,105], hepatocellular carcinoma [30], laryngeal squamous cell carcinoma [31], lung cancer [32], neuroblastoma [33], pancreatic cancer [34], and prostate cancer. HK2 expression in these cancers inversely correlates to overall patient survival rates [35]. The genetic ablation of HK2 is known to inhibit malignant growth in mouse models [36,106,107,108,109]. A landmark study on an adult tumor model of mice demonstrated the therapeutic effects of systemic deletion of HK2 [36,115,116,117,118]. In addition to its enzymatic activity, the mitochondrial binding ability of HK2 plays a role in inhibiting apoptosis and upregulating synthetic pathways which support tumor growth (Figure 2). The mitochondrial-bound HK2 is therefore elevated in many forms of cancer [37,38,39]. The amplification of HK2 appears to be related to the expression of p53. Recent studies have shown that p53-inducible protein TIGAR (Tp53-induced glycolysis and apoptosis regulator), Akt, and ER stress sensor kinase could regulate mitochondrial HK2 localization [32,117,119,120,121,122,123]. Interestingly, the mitochondrial TIGAR–HK2 complex upregulated HK2 and hypoxia-inducible factor 1 (HIF1) activity, which limits reactive oxygen species (ROS) production and protects against tumor cell death under hypoxic conditions [124,125,126,127,128,129]. It is also observed that the GCK to HK2 switch occurs in hepatocellular carcinoma (HCC), and the expression of HK2 is highest in HCC [127]. Additionally, HK2 is also regulated by epigenetic mediators, including long non-coding RNAs [38,39,106,107], microRNAs [123,124,125,126,127], histone, and DNA methylation [109]. HK2 is localized to the outer mitochondrial membrane through a voltage-dependent anion channel (VDAC) [126] (Figure 2). This association permits direct access to the ATP generated within the mitochondria [124]. This phenomenon is especially significant in malignant cells where rates of aerobic glycolysis go up tremendously to meet the energy demands of the transformed cell (Warburg effect) [105].

*Hexokinase 3:* HK3 is upregulated in several cancers, including acute myeloid leukemia (AML), where it plays the role of an anti-apoptotic protein to promote tumor cell survival alongside HK1 and 2 [130,131]. The previously identified functions of the enzyme include cell survival through the attenuation of apoptosis and the enhancement of mitochondrial biogenesis [2,132,133]. The latest research about the functions of HK3 in normal and cancer cells has uncovered previously unanticipated roles of this protein. A recent study by Seiler et al. has reported that hexokinase 3 enhances myeloid cell survival via non-glycolytic functions [134]. In contrast, another report by Xu et al. showed that HK3 dysfunction promotes tumorigenesis and immune escape by upregulating macrophage infiltration in renal cell carcinoma [135].

*Glucokinase:* Glucose phosphorylation activity for GCK has been observed in several cancer cell lines [136]. GCK is also known to interact with BAD (Bcl-2 agonist of cell death) to integrate glycolysis with apoptosis [50,137,138,139]. To date, 17 activating mutations targeted by multiple activators have been identified in the allosteric activator site of GCK [140,141,142,143]. The activating variations and their targeting by the activators lead to enhanced cellular proliferation, including the proliferation of cancer cell lines such as INS, which indicates a putative pro-oncogenic role for GCK [144,145,146]. Although there is no direct evidence for the role of GCK as a pro-oncogene, recent reports exploring somatic variations of allosterically regulated proteins in cancer genomes suggest that somatic mutations of GCK could play a role in tumorigenesis [147]. Těšínský et al. provide the first direct evidence of the role of GCK in tumorigenesis by demonstrating a change in the kinetic properties of GCK which include an increased affinity for glucose and changes in cooperative binding [148].

**Figure 3:**
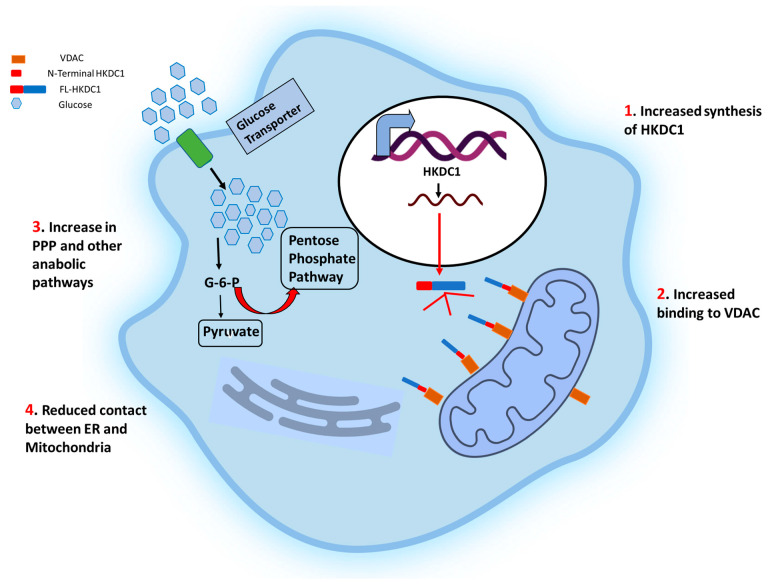
**Schematic representation of the effects of HKDC1 over-expression in cancer cells.** The cell membrane glucose transporters (GLUT 1/3) mediate the glucose uptake, which is degraded to pyruvate by glycolysis. Upregulation of HKDC1 (and other HKs) in many cancer types leads to enhanced generation of glycolytic intermediate, which functions as precursors for numerous metabolic pathways necessary for the biosynthesis of cellular components; pentose phosphate pathway (marked with thick red arrows), cholesterol biosynthesis, and fatty acid biosynthesis. Notably, HKDC1 upregulation leads to an increase in HKDC1-mitochondrial binding, which is responsible for the maintenance of glycolysis and TCA cycle and contributes to unabated cell proliferation through the aversion of apoptosis and endoplasmic reticulum (ER)-mediated stress response mechanisms by reducing the number of physical contact points between ER and mitochondria.

*Hexokinase domain containing 1:* Studies performed over the past decade have linked HKDC1 to various functions (Figure 3). Much of the interest in HKDC1’s role in cancer stems from the fact that, like HK1 and 2, it localizes in the mitochondrial outer membrane (MOM) and binds with the voltage-dependent anion channel (VDAC) [14]. We were the first to identify the role of hepatic HKDC1 in glucose metabolism. Using a mouse model of HKDC1, we demonstrated that hepatic HKDC1 modulates glucose metabolism and insulin sensitivity in mice. Although HKDC1 has nominal expression in normal hepatocytes [17], it is significantly upregulated in hepatocellular carcinoma (HCC) cells [149,150], implying that it plays an essential role in HCC. By using HKDC1 knockout models, we have shown that cellular HK activity is not affected by HKDC1 ablation; however, there is a significant increase in glucose uptake, where the bulk of glucose carbons flow through the glycolytic shunt pathways PPP and HBP (Figure 3) [16]. We further show that HKDC1 interacts with the mitochondria, and its loss results in mitochondrial dysfunction [16]. Since cancer cells require ATP to prepare for cell division during the synthetic (S) phase of the cell cycle, a deficiency in ATP may cause cell cycle arrest. Others have shown that HKDC1 is also significantly increased in breast cancer cells, enhancing glucose uptake and mitochondrial membrane potential to encourage cell survival and growth. In agreement with this phenomenon, HKDC1 knockdown increased the production of reactive oxygen species (ROS), the activation of caspase 3, and apoptosis [52]. Li et al. [151] used RNA-seq data from The Cancer Genome Atlas to pinpoint genetically altered genes in a univariate survival analysis of patients with squamous cell lung carcinoma (SQCLC). Seven thousand two hundred twenty-two genetically modified genes were discovered by the analysis of RNA-seq data from 550 SQCLC patients, and HKDC1 was one of 14 feature genes with more than 100 frequencies linked to a worse prognosis [151,152]. HKDC1 mRNA and protein levels were also expressed higher in lung cancer cell lines than in healthy lung epithelial cells.

Additionally, there was a direct correlation between the degree of HKDC1 protein expression and histological differentiation, reduced survival, tumor size, pN (N refers to the number of nearby lymph nodes with cancer) stage, and poor prognosis. In agreement with these results, lung cancer cell lines stably overexpressing HKDC1 demonstrated increased glucose consumption, lactate generation, proliferation, migration, and invasion compared to healthy lung epithelial cells [151,152]. A comparison study on RNA sequencing (RNA-Seq) analysis of colorectal cancer (CRC) and matched standard tissue samples has observed significant splicing variations in nine genes in CRC. Interestingly, the authors discovered alternate regulation of the first exon in HKDC1 using exon sequencing (DEXSeq) to uncover variations in relative exon usage. HKDC1 E1a-E3a was elevated in CRC, suggesting a potential functional impact because of a projected change in the HKDC1 protein sequence [153,154,155]. Another study has reported a 13 h phase change in HKDC1 expression between SW480 cells and their metastatic counterpart SW620 (a core clock gene) that occurs in conjunction with a phase shift in aryl hydrocarbon receptor nuclear translocator-like protein-1 (BMAL1). In SW480 cells, silencing BMAL1 results in an elevation of HKDC1 expression, and this effect was eliminated in SW620 cells. These findings imply that HKDC1 and the circadian clock interact, as the circadian clock is altered in metastatic cells [156].

Eukaryotic cells adjust to cellular stress by phosphorylating eukaryotic translation initiation factor 2 alpha (eIF2), which results in the translation of specific transcripts that enable the cell to withstand stress [123,124,125,126,157,158]. Activating transcription factor 4 (ATF4) is a leucine zipper transcription factor that modulates the cellular integrated stress response to allow cells to adapt to and endure stressors [133,159,160]. The overexpression of ATF4 causes the HKDC1 gene transcription to increase significantly under cellular stress, changing hepatocyte mitochondrial dynamics [161]. HKDC1 is upregulated in response to the endoplasmic reticulum (ER) stress or mitochondrial respiratory chain inhibition; however, when these stressors are present in combination with RNA interference to decrease ATF4, HKDC1 gene expression is reduced [161].

## 2. Future Directions

Accelerated aerobic glycolysis is a hallmark of cancer cells which provides a rapid source of ATP and good metabolic intermediates for synthesizing nucleic acids, lipids, and proteins in the rapidly dividing cells [162,163]. The increased dependency of cancer cells on glucose metabolism sets them apart from their regular counterparts and could render them more vulnerable to disruption in glucose metabolism. Cancer cells could therefore be selectively targeted through the disruption of glucose metabolism, and the therapeutic targeting of HK enzymes in cancers has seemed to be a plausible strategy. However, considering the overarching redundancy in the catalytic activity of different isozymes, it seems reasonable to argue that one isoform could compensate for another under specified conditions. A lack of literature on the non-redundant functions of each isozyme further complicates this approach. The therapeutic targeting of HKs in cancer per se awaits more targeted approaches for effective outcomes. Identifying isoform-specific roles in cancer could reveal more selective targets that could be utilized for therapeutic purposes without compromising overall homeostasis.

HK1-2 and HKDC1 contain a mitochondrial binding site in the N-terminal domain. This domain mediates HK1 activity in normal cells while it plays a role in tumorigenesis in HK2 and HKDC1 [21,43,164]. HK2 is known to inhibit apoptosis and regulate autophagy [27]. The recent identification of HK2 localization to contact points between mitochondria and endoplasmic reticulum, known as mitochondria-associated membranes (MAMs), has unveiled a novel role of HK2 in regulating Ca^2+^ flux within the cells [165,166]. HKDC1 is also postulated to bind to MAMs similarly and regulate Ca^2+^ flux. In the future, the binding of HK2 and HKDC1 could be specifically targeted as a promising therapeutic strategy for effective outcomes in cancer. Of particular interest, small molecular inhibitors which specifically target the binding of HK2 and HKDC1 to mitochondria and MAMs need further exploration [167]. Such inhibitors have recently been characterized for HK2, which specifically and selectively target HK2 without producing off-target effects. Evaluating similar inhibitors for HKDC1 could prove to be an effective therapeutic avenue for cancer treatment in the future [167]. 

## Figures and Tables

**Table 1:** Characteristics of HK isoforms in humans.

	HK1	HK2	HK3	GCK	HKDC1
**Gene location (Human)**	10q22	2p13	5q35.2	7p15.1	10q22
**MW (kDa)**	~100	~100	~100	~50	~100
**Number of catalytic domains**	1	2	1	1	1
**Km for glucose** (mmol L^−1^)	0.03	0.3	0.003	6	-
**Km for ATP** (mmol L^−1^)	0.5	0.7	1.0	0.6	-
**G6P inhibition** *K*_i_ (mmol L^−1^)	0.02	0.02	0.10	-	-
**Effect of Pi**	Low conc counteracts G6P inhibition, but high conc is inhibitory	inhibitory	Inhibitory	-	-
**Insulin regulation**	-	+	*	+	*
**Major tissue expression**	Brain, Kidney	Muscle, adipose	Lung, spleen	Liver, pancreas	GI, Kidney, and Brain
**Mitochondrial binding**	✓	✓	✕	✕	✓

+ = effect; - = NO effect; * = Sufficient data not available; Pi = inorganic phosphate; ✓ = binding; ✕ = no binding.

## Data Availability

Data used in this manuscript is publicly available at https://www.cancer.gov/ccg/research/genome-sequencing/tcga accessed on 10 March 2023.

## Supplementary Materials

The following supporting information can be downloaded at: https://www.mdpi.com/article/10.3390/life13040946/s1, Figure S1: Protein sequence alignment of hexokinase isoforms; Figure S2: Differential expression of hexokinase isoforms; Table S1: TCGA study abbreviations.

## References

[1] Warburg O. (1956). On the origin of cancer cells. Science.

[2] Liberti M.V., Locasale J.W. (2016). The Warburg effect: How does it benefit cancer cells?. Trends Biochem. Sci..

[3] Katzen H.M., Schimke R.T. (1965). Multiple forms of hexokinase in the rat: Tissue distribution, age dependency, and properties. Proc. Natl. Acad. Sci. USA.

[4] Sebastian S., Hoebee B., Hande M., Kenkare U., Natarajan A. (1997). Assignment of hexokinase types 1, 2, 3 (Hk1, 2, 3) and glucokinase (Gck) to rat chromosome band 20q11, 4q34, 17q12 and 14q21 respectively, by in situ hybridization. Cytogenet. Cell Genet..

[5] Wilson J.E. (2003). Isozymes of mammalian hexokinase: Structure, subcellular localization and metabolic function. J. Exp. Biol..

[6] Matschinsky F.M., Wilson D.F. (2019). The central role of glucokinase in glucose homeostasis: A perspective 50 years after demonstrating the presence of the enzyme in islets of Langerhans. Front. Physiol..

[7] Martínez-Reyes I., Chandel N.S. (2021). Cancer metabolism: Looking forward. Nat. Rev. Cancer.

[8] Mathupala S.P., Ko Y.H., Pedersen P.L. (2006). Hexokinase II: Cancer’s double-edged sword acting as both facilitator and gatekeeper of malignancy when bound to mitochondria. Oncogene.

[9] John S., Weiss J.N., Ribalet B. (2011). Subcellular localization of hexokinases I and II directs the metabolic fate of glucose. PLoS ONE.

[10] Wyatt E., Wu R., Rabeh W., Park H.W., Ghanefar M., Ardehali H. (2010). Regulation and cytoprotective role of hexokinase III. PLoS ONE.

[11] Cárdenas M.L., Cornish-Bowden A., Ureta T. (1998). Evolution and regulatory role of the hexokinases. Biochim. Biophys. Acta Mol. Cell Res..

[12] Postic C., Shiota M., Magnuson M.A. (2001). Cell-specific roles of glucokinase in glucose homeostasis. Recent Prog. Hormone Res..

[13] Ludvik A.E., Pusec C.M., Priyadarshini M., Angueira A.R., Guo C., Lo A., Hershen-house K.S., Yang G.Y., Ding X., Reddy T.E. (2016). Is a novel hexokinase involved in whole-body glucose use. Endocrinology.

[14] Pusec C.M., De Jesus A., Khan M.W., Terry A.R., Ludvik A.E., Xu K., Giancola N., Pervaiz H., Smith E.D., Ding X. (2019). Hepatic HKDC1 expression contributes to liver metabolism. Endocrinology.

[15] Khan M.W., Priyadarshini M., Cordoba-Chacon J., Becker T.C., Layden B.T. (2019). Hepatic hexokinase domain containing 1 (HKDC1) improves whole body glucose tolerance and insulin sensitivity in pregnant mice. Biochim. Biophys. Acta (BBA) Mol. Basis Dis..

[16] Khan M.W., Terry A.R., Priyadarshini M., Ilievski V., Farooq Z., Guzman G., Cordoba-Chacon J., Ben-Sahra I., Wicksteed B., Layden B.T. (2022). The hexokinase “HKDC1” interaction with the mitochondria is essential for liver cancer progression. Cell Death Dis..

[17] Khan M.W., Ding X., Cotler S.J., Clarke M., Layden B.T. (2018). Studies on the tissue localization of HKDC1, a putative novel fifth hexokinase, in humans. J. Histochem. Cytochem..

[18] Zapater J.L., Lednovich K.R., Khan M.W., Pusec C.M., Layden B.T. (2022). Hexokinase domain-containing protein-1 in metabolic diseases and beyond. Trends Endocrinol. Metab..

[19] Hayes M.G., Urbanek M., Hivert M.F., Armstrong L.L., Morrison J., Guo C., Lowe L.P., Scheftner D.A., Pluzhnikov A., Levine D.M. (2013). Identification of HKDC1 and BACE2 as genes influencing glycemic traits during pregnancy through genome-wide association studies. Diabetes.

[20] Irwin D.M., Tan H. (2008). Molecular evolution of the vertebrate hexokinase gene family: Identification of a conserved fifth vertebrate hexokinase gene. Comp. Biochem. Physiol. Part D Genomics Proteomics.

[21] Guo C., Ludvik A.E., Arlotto M.E., Hayes M.G., Armstrong L.L., Scholtens D.M. (2015). Coordinated regulatory variation associated with gestational hyperglycemia regulates expression of the novel hexokinase HKDC1. Nat. Commun..

[22] Colowick S.P., Boyer P.D. (1973). The hexokinases. The Enzymes.

[23] Easterby J.S., O’Brien M.J. (1973). Purification and properties of pig-heart hexokinase. Eur. J. Biochem..

[24] White T.K., Wilson J.E. (1989). Isolation and characterization of the discrete N- and C-terminal halves of rat brain hexokinase: Retention of full catalytic activity in the isolated C-terminal half. Arch. Biochem. Biophys..

[25] Arora K.K., Filburn C.R., Pedersen P.L. (1993). Structure/function relationships in hexokinase. Site-directed mutational analyses and characterization of overexpressed fragments implicate different functions for the N- and C-terminal halves of the enzyme. J. Biol. Chem..

[26] Ureta T., Medina C., Preller A. (1987). The evolution of hexokinases. Arch. Biol. Med. Exp..

[27] Tsai H.J. (1999). Functional organization and evolution of mammalian hexokinases: Mutations that caused the loss of catalytic activity in N-terminal halves of type I and type III isozymes. Arch. Biochem. Biophys..

[28] Kawai S. (2005). Hypothesis: Structures, evolution, and ancestor of glucose kinases in the hexokinase family. J. Biosci. Bioeng..

[29] Heikkinen S., Suppola S., Malkki M., Deeb S.S. (2000). Mouse hexokinase II gene: Structure, cDNA, promoter analysis, and expression pattern. Mamm. Genome.

[30] Kwee S.A., Hernandez B., Chan O., Wong L. (2012). Choline kinase alpha and hexokinase-2 protein expression in hepatocellular carcinoma: Association with survival. PLoS ONE.

[31] Chen J., Zhang S., Li Y., Tang Z., Kong W. (2014). Hexokinase 2 overexpression promotes the proliferation and survival of laryngeal squamous cell carcinoma. Tumor Biol..

[32] Wang H., Wang L., Zhang Y., Wang J., Deng Y., Lin D. (2016). Inhibition of glycolytic enzyme hexokinase II (HK2) suppresses lung tumor growth. Cancer Cell Int..

[33] Botzer L.E., Maman S., Sagi-Assif O., Meshel T., Nevo I., Yron I., Witz I.P. (2016). Hexokinase 2 is a determinant of neuroblastoma metastasis. Br. J. Cancer.

[34] Ogawa H., Nagano H., Konno M., Eguchi H., Koseki J., Kawamoto K., Nishida N., Colvin H., Tomokuni A., Tomimaru Y. (2015). The combination of the expression of hexokinase 2 and pyruvate kinase M2 is a prognostic marker in patients with pancreatic cancer. Mol. Clin. Oncol..

[35] He H.C., Bi X.C., Zheng Z.W., Dai Q.S., Han Z.D., Liang Y.X., Ye Y.K., Zeng G.H., Zhu G., Zhong W.D. (2009). Real-time quantitative RT-PCR assessment of PIM-1 and hK2 mRNA expression in benign prostate hyperplasia and prostate cancer. Med. Oncol..

[36] Patra K.C., Wang Q., Bhaskar P.T., Miller L., Wang Z., Wheaton W., Chandel N., Laakso M., Muller W.J., Allen E.L. (2013). Hexokinase 2 is required for tumor initiation and maintenance and its systemic deletion is therapeutic in mouse models of cancer. Cancer Cell.

[37] Bacci M., Giannoni E., Fearns A., Ribas R., Gao Q., Taddei M.L., Pintus G., Dowsett M., Isacke C.M., Martin L.A. (2016). miR-155 drives metabolic reprogramming of ER+ breast cancer cells following long-term estrogen deprivation and predicts clinical response to aromatase inhibitors. Cancer Res..

[38] van ‘t Veer L.J., Dai H., van de Vijver M.J., He Y.D., Hart A.A.M., Mao M., Peterse H.L., van der Kooy K., Marton M.J., Witteveen A.T. (2002). Gene expression profiling predicts clinical outcome of breast cancer. Nature.

[39] Liu X., Miao W., Huang M., Li L., Dai X., Wang Y. (2019). Elevated hexokinase II expression confers acquired resistance to 4-hydroxytamoxifen in breast cancer cells. Mol. Cell Proteomics.

[40] Palmieri D., Fitzgerald D., Shreeve S.M., Hua E., Bronder J.L., Weil R.J., Davis S., Stark A.M., Merino M.J., Kurek R. (2009). Analyses of resected human brain metastases of breast cancer reveal the association between up-regulation of hexokinase 2 and poor prognosis. Mol. Cancer Res..

[41] Federzoni E.A., Humbert M., Torbett B.E., Behre G., Fey M.F., Tschan M.P. (2014). CEBPA-dependent HK3 and KLF5 expression in primary AML and during AML differentiation. Sci. Rep..

[42] Franz M.M. (2005). Glucokinase, glucose homeostasis, and diabetes mellitus. Curr. Diab. Rep..

[43] Iynedjian P.B. (1993). Mammalian glucokinase and its gene. Biochem. J..

[44] Ferre T., Riu E., Bosch F., Valera A. (1996). Evidence from transgenic mice that glucokinase is rate limiting for glucose utilization in the liver. FASEB J..

[45] Wilson J.E. (1995). Hexokinases. Rev. Physiol. Biochem. Pharmacol..

[46] Matschinsky F.M. (1990). Glucokinase as glucose sensor and metabolic signal generator in pancreatic β-cells and hepatocytes. Diabetes.

[47] Velho G., Froguel P., Clement K., Pueyo M.E., Rakotoambinina B., Zouali H., Passa P., Cohen D., Robert J.J. (1992). Primary pancreatic beta-cell secretory defect caused by mutations in glucokinase gene in kindreds of maturity onset diabetes of the young. Lancet.

[48] Byrne M.M., Sturis J., Clement K., Vionnet N., Pueyo M.E., Stoffel M., Takeda J., Passa P., Cohen D., Bell G.I. (1994). Insulin secretory abnormalities in subjects with hyperglycemia due to glucokinase mutations. J. Clin. Investig..

[49] Velho G., Petersen K.F., Perseghin G., Hwang J.H., Rothman D.L., Pueyo M.E., Cline G.W., Froguel P., Shulman G.I. (1996). Impaired hepatic glycogen synthesis in glucokinase-deficient (MODY-2) subjects. J. Clin. Investig..

[50] Danial N.N., Gramm C.F., Scorrano L., Zhang C.Y., Krauss S., Ranger A.M., Datta S.R., Greenberg M.E., Licklider L.J., Lowell B.B. (2003). BAD and glucokinase reside in a mitochondrial complex that integrates glycolysis and apoptosis. Nature.

[51] Danial N.N., Walensky L.D., Zhang C.Y., Choi C.S., Fisher J.K., Molina A.J., Datta S.R., Pitter K.L., Bird G.H., Wikstrom J.D. (2008). Dual role of proapoptotic BAD in insulin secretion and beta cell survival. Nat. Med..

[52] Chen X., Lv Y., Sun Y., Zhang H., Xie W., Zhong L., Chen Q., Li M., Li L., Feng J. (2019). PGC1β regulates breast tumor growth and metastasis by SREBP1-mediated HKDC1 expression. Front. Oncol..

[53] Chen Q., Feng J., Wu J., Yu Z., Zhang W., Chen Y., Yao P., Zhang H. (2020). HKDC1 C-terminal based peptides inhibit extranodal natural killer/T-cell lymphoma by modulation of mitochondrial function and EBV suppression. Leukemia.

[54] Ahn K.J., Kim J., Yun M., Park J.H., Lee J.D. (2009). Enzymatic properties of the N- and C-terminal halves of human hexokinase, II. BMB Rep..

[55] Demetrius L., Tuszynski J.A. (2010). Quantum metabolism explains the allometric scaling of metabolic rates. J. R. Soc. Interface.

[56] Printz R.L., Koch S., Potter L.R., O’Doherty R.M., Tiesinga J.J., Moritz S., Granner D.K. (1993). Hexokinase II mRNA and gene structure, regulation by insulin, and evolution. J. Biol. Chem..

[57] White J.A., Liu W., Wilson J.E. (1996). Isolation of the promoter for Type I hexokinase from rat. Arch. Biochem. Biophys..

[58] Liu W., Wilson J.E. (1997). Two Sp sites are important cis elements regulating the upstream promoter region of the gene for rat Type I hexokinase. Arch. Biochem. Biophys..

[59] Mathupala S.P., Rempel A., Pedersen P.L. (1995). Glucose catabolism in cancer cells. Isolation, sequence, and activity of the promoter for Type II hexokinase. J. Biol. Chem..

[60] Osawa H., Robey R.B., Printz R.L., Granner D.K. (1996). Identification and characterization of basal and cyclic AMP response elements in the promoter of the rat hexokinase II gene. J. Biol. Chem..

[61] Sebastian S., White J.A., Wilson J.E. (1999). Characterization of the rat Type III hexokinase gene promoter. A functional octamer 1 motif is critical for basal promoter activity. J. Biol. Chem..

[62] Sebastian S., Edassery S., Wilson J.E. (2001). The human gene for the Type III isozyme of hexokinase. Structure, basal promoter, and evolution. Arch. Biochem. Biophys..

[63] Heikkinen S., Pietilä M., Halmekytö M., Suppola S., Pirinen E., Deeb S.S., Jänne J., Laakso M. (1999). Hexokinase II-deficient mice. Prenatal death of homozygotes without disturbances in glucose tolerance in heterozygotes. J. Biol. Chem..

[64] Murakami K., Kanno H., Tancabelic J., Fujii H. (2002). Gene expression and biological significance of hexokinase in erythroid cells. Acta Haematol..

[65] Wakeling M.N., Owens N.D.L., Hopkinson J.R., Johnson M.B., Houghton J.A.L., Dasta-mani A., Flaxman C.S., Wyatt R.C., Hewat T.I., Hopkins J.J. (2022). Non-coding variants disrupting a tissue-specific regulatory element in HK1 cause congenital hyperinsulinism. Nat. Genet..

[66] Feriotto G., Finotti A., Breveglieri G., Treves S., Zorzato F., Gambari R. (2007). Transcriptional activity and Sp 1/3 transcription factor binding to the P1 promoter sequences of the human AbetaH-J-J locus. FEBS J..

[67] Liu B., Qu X., Wang J., Xu L., Zhang L., Xu B., Su J., Bian X. (2023). LINC00365 functions as a tumor suppressor by inhibiting HIF-1α-mediated glucose metabolism reprogramming in breast cancer. Exp. Cell Res..

[68] Kim H.R., Roe J.S., Lee J.E., Cho E.J., Youn H.D. (2013). p53 regulates glucose metabolism by miR-34a. Biochem. Biophys. Res. Commun..

[69] Tong A.W., Nemunaitis J. (2008). Modulation of miRNA activity in human cancer: A new paradigm for cancer gene therapy?. Cancer Gene Ther..

[70] Roberts D.J., Miyamoto S. (2015). Hexokinase II integrates energy metabolism and cellular protection: Akting on mitochondria and TORCing to autophagy. Cell Death Differ..

[71] Bustamante E., Morris H.P., Pedersen P.L. (1981). Energy metabolism of tumor cells. Requirement for a form of hexokinase with a pro-pensity for mitochondrial binding. J. Biol. Chem..

[72] Rempel A., Mathupala S.P., Griffin C.A., Hawkins A.L., Pedersen P.L. (1996). Glucose catabolism in cancer cells: Amplification of the gene encoding type II hexokinase. Cancer Res..

[73] Mathupala S.P., Rempel A., Pedersen P.L. (1997). Aberrant glycolytic metabolism of cancer cells: A remarkable coordination of genetic, transcriptional, post-translational, and mutational events that lead to a critical role for type II hexokinase. J. Bioenerg. Biomembr..

[74] Mayer D., Klimek F., Rempel A., Bannasch P. (1997). Hexokinase expression in liver preneoplasia and neoplasia. Biochem. Soc. Trans..

[75] Pedersen P.L., Mathupala S., Rempel A., Geschwind J.F., Ko Y.H. (2002). Mitochondrial bound type II hexokinase: A key player in the growth and survival of many cancers and an ideal prospect for therapeutic intervention. Biochim. Biophys. Acta.

[76] Miyamoto S., Murphy A.N., Brown J.H. (2008). Akt mediates mitochondrial protection in cardiomyocytes through phosphorylation of mitochondrial hexokinase-II. Cell Death Differ..

[77] Katzen H.M. (1966). The effect of diabetes and insulin in vivo and in vitro on a low Km form of hexokinase from various rat tissues. Biochem. Biophys. Res. Commun..

[78] Katzen H.M., Soderman D.D., Wiley C.E. (1970). Multiple forms of hexokinase. Activities associated with subcellular particulate and soluble fractions of normal and streptozotocin diabetic rat tissues. J. Biol. Chem..

[79] Burcelin R., Printz R.L., Kande J., Assan R., Granner D.K., Girard J. (1993). Regulation of glucose transporter and hexokinase II expression in tissues of diabetic rats. Am. J. Physiol..

[80] Gurel E., Ustunova S., Kapucu A., Yilmazer N., Eerbeek O., Nederlof R., Hollmann M.W., Demirci-Tansel C., Zuurbier C.J. (2013). Hexokinase cellular trafficking in ischemia-reperfusion and ischemic preconditioning is altered in type I diabetic heart. Mol. Biol. Rep..

[81] Sebastian S., Horton J.D., Wilson J.E. (2000). Anabolic function of the Type II isozyme of hexokinase in hepatic lipid synthesis. Biochem. Biophys. Res. Commun..

[82] Kaselonis G.L., McCabe E.R., Gray S.M. (1999). Expression of hexokinase 1 and hexokinase 2 in mammary tissue of nonlactating and lactating rats: Evaluation by RT-PCR. Mol. Genet. Metab..

[83] Osbak K.K., Colclough K., Saint-Martin C., Beer N.L., Bellanné-Chantelot C., Ellard S., Gloyn A.L. (2009). Update on mutations in glucokinase (GCK), which cause maturity-onset diabetes of the young, permanent neonatal diabetes, and hyperinsulinemic hypoglycemia. Hum. Mutat..

[84] Iynedjian P.B. (2009). Molecular physiology of mammalian glucokinase. Cell Mol. Life Sci..

[85] Moates J.M., Nanda S., Cissell M.A., Tsai M.-J., Stein R. (2003). BETA2 activates transcription from the upstream glucokinase gene promoter in islet β-cells and gut endocrine cells. Diabetes.

[86] Jetton T.L., Liang Y., Pettepher C.C., Zimmerman E.C., Cox F.G., Horvath K., Matschinsky F.M., Magnuson M.A. (1994). Analysis of up-stream glucokinase promoter activity in transgenic mice and identification of glucokinase in rare neuroendocrine cells in the brain and gut. J. Biol. Chem..

[87] Moates J.M., Magnuson M.A. (2004). The Pal elements in the upstream glucokinase promoter exhibit dyad symmetry and display cell-specific enhancer activity when multimerized. Diabetologia.

[88] Sternisha S.M., Miller B.G. Molecular and Cellular Regulation of Human Glucokinase. Arch. Biochem. Biophys..

[89] Peter A., Stefan N., Cegan A., Walenta M., Wagner S., Königsrainer A., Königsrain-er A., Machicao F., Schick F., Häring H.-U. (2011). Hepatic Glucokinase Expression Is Associated with Lipogenesis and Fatty Liver in Humans. J. Clin. Endocrinol. Metab..

[90] Iynedjian P.B., Gjinovci A., Renold A.E. (1988). Stimulation by insulin of glucokinase gene transcription in liver of diabetic rats. J. Biol. Chem..

[91] Wu C., Okar D.A., Stoeckman A.K., Peng L.J., Herrera A.H., Herrera J.E., Towle H.C., Lange A.J. (2004). A potential role for fructose-2,6-bisphosphate in the stimulation of hepatic glucokinase gene expression. Endocrinology.

[92] Roth U., Curth K., Unterman T.G., Kietzmann T. (2004). The transcription factors HIF-1 and HNF-4 and the coactivator p300 are involved in insulin-regulated glucokinase gene expression via the phosphatidylinositol 3-kinase/protein kinase B pathway. J. Biol. Chem..

[93] Iynedjian P.B., Marie S., Gjinovci A., Genin B., Deng S.P., Buhler L., Morel P., Mentha G. (1995). Glucokinase and cytosolic phosphoenolpyruvate carboxykinase (GTP) in the human liver. Regulation of gene expression in cultured hepatocytes. J. Clin. Investig..

[94] Agius L. (2016). Hormonal and Metabolite Regulation of Hepatic Glucokinase. Annu. Rev. Nutr..

[95] Stubbs M., Aiston S., Agius L. (2000). Subcellular localization, mobility, and kinetic activity of glucokinase in glucose-responsive insulin-secreting cells. Diabetes.

[96] Preller A andWilson J.E. (1992). Localization of the type III isozyme of hexokinase at the nuclear periphery. Arch. Biochem. Biophys..

[97] Guillaume C., Bernard R., Scott J., Paavo K., Peipei P., James N.W. (2015). Hexokinases and cardioprotection. J. Mol. Cell Cardiol..

[98] Nishizawa T., Kanter J.E., Kramer F., Barnhart S., Shen X., Vivekanandan-Giri A., Wall V.Z., Kowitz J., Devaraj S., O’Brien K.D. (2014). Testing the role of myeloid cell glucose flux in inflammation and atherosclerosis. Cell Rep..

[99] Moon J.S., Hisata S., Park M.A., DeNicola G.M., Ryter S.W., Nakahira K., Choi AMK (2015). mTORC1-induced HK1-dependent glycolysis regulates NLRP3 inflammasome activation. Cell Rep..

[100] De Jesus A., Keyhani-Nejad F., Pusec C.M., Goodman L., Geier J.A., Stoolman J.S., Stanczyk P.J., Nguyen T., Xu K., Suresh K.V. (2022). Hexokinase 1 cellular localization regulates the metabolic fate of glucose. Mol. Cell..

[101] van der Kooij M.A., Rojas-Charry L., Givehchi M., Wolf C., Bueno D., Arndt S., Ten-zer S., Mattioni L., Treccani G., Hasch A. (2022). Chronic social stress disrupts the intracellular redistribution of brain hexokinase 3 induced by shifts in peripheral glucose levels. J. Mol. Med..

[102] Huang J.B., Kindzelskii A.L., Petty H.R. (2002). Hexokinase translocation during neutrophil activation, chemotaxis, and phagocytosis: Disruption by cytochalasin, D.; dexamethasone, and indomethacin. Cell. Immunol..

[103] Labrecque M.P., Brown L.G., Coleman I.M., Nguyen H.M., Lin D.W., Corey E., Nelson P.S., Morrissey C. (2021). Cabozantinib can block growth of neuroendocrine prostate cancer patient-derived xenografts by disrupting tumor vasculature. PLoS ONE.

[104] Lee H.G., Kim H., Son T., Jeong Y., Kim S.U., Dong S.M., Park Y.N., Lee J.D., Lee J.M., Park J.H. (2016). Regulation of HK2 expression through alterations in CpG methylation of the HK2 promoter during progression of hepatocellular carcinoma. Oncotarget.

[105] Sun L., Shukair S., Naik T.J., Moazed F., Ardehali H. (2008). Glucose phosphorylation and mitochondrial binding are required for the protective effects of hexokinases I and, II. Mol. Cell Biol..

[106] Wilson J.E., Beitner R. (1985). Regulation of mammalian hexokinase activity. Regulation of Carbohydrate Metabolism.

[107] Li Y., Tian H., Luo H., Fu J., Jiao Y., Li Y. (2020). Prognostic significance and related mechanisms of hexokinase 1 in ovarian cancer. Onco Targets Ther..

[108] Xu S., Catapang A., Doh H.M., Bayley N.A., Lee J.T., Braas D., Graeber T.G., Herschman H.R. (2019). Hexokinase 2 is targetable for HK1 negative, HK2 positive tumors from a wide variety of tissues of origin. J. Nucl. Med..

[109] Xu S., Catapang A., Braas D., Stiles L., Doh H.M., Lee J.T., Graeber T.G., Damoiseaux R., Shirihai O., Herschman H.R. (2018). A precision therapeutic strategy for hexokinase 1-null, hexokinase 2-positive cancers. Cancer Metab..

[110] Šimčíková D., Gardáš D., Hložková K., Hruda M., Žáček P., Rob L., Heneberg P. (2021). Loss of hexokinase 1 sensitizes ovarian cancer to high-dose metformin. Cancer Metab..

[111] Amendola C.R., Mahaffey J.P., Parker S.J., Ahearn I.M., Chen W.C., Zhou M., Court H., Shi J., Mendoza S.L., Morten M.J. (2019). KRAS4 directly regulates HK1. Nature.

[112] Yang X., Cheng Y., Li P., Tao J., Deng X., Zhang X., Gu M., Lu Q., Yin C. (2015). A lentiviral sponge for miRNA-21 diminishes aerobic glycolysis in bladder cancer T24 cells via the PTEN/PI3K/AKT/mTOR axis. Tumour Biol..

[113] Huang X., Liu M., Sun H., Wang F., Xie X., Chen X., Su J., He Y., Dai Y., Wu H. (2015). HK2 is a radiation resistant and independent negative prognostic factor for patients with locally advanced cervical squamous cell carcinoma. Int. J. Clin. Exp. Pathol..

[114] Iwamoto M., Kawada K., Nakamoto Y., Itatani Y., Inamoto S., Toda K., Kimura H., Sasazuki T., Shirasawa S., Okuyama H. (2014). Regulation of ^18F-FDG accumulation in colorectal cancer cells with mutated KRAS. J. Nucl. Med..

[115] Christofk H.R., Vander Heiden M.G., Harris M.H., Ramanathan A., Gerszten R.E., Wei R., Fleming M.D., Schreiber S.L., Cantley L.C. (2008). The M2 splice isoform of pyruvate kinase is important for cancer metabolism and tumour growth. Nature.

[116] Marybeth A., Raoud M., Richard M., Jen J.Y. (2016). Hexokinase 2 promotes tumor growth and metastasis by regulating lactate production in pancreatic cancer. Oncotarget.

[117] Wang L., Xiong H., Wu F., Zhang Y., Wang J., Zhao L., Guo X., Chang L.J., Zhang Y., You M.J. (2014). Hexokinase 2-mediated Warburg effect is required for PTEN- and p53-deficiency-driven prostate cancer growth. Cell Rep..

[118] Wolf A., Agnihotri S., Micallef J., Mukherjee J., Sabha N., Cairns R., Hawkins C., Guha A. (2011). Hexokinase 2 is a key mediator of aerobic glycolysis and promotes tumor growth in human glioblastoma multiforme. J. Exp. Med..

[119] Semenza G.L. (2010). HIF-1: Upstream and downstream of cancer metabolism. Curr. Opin. Genet. Dev..

[120] Cheung E.C., Ludwig R.L., Vousden K.H. (2012). Mitochondrial localization of TIGAR under hypoxia stimulates HK2 and lowers ROS and cell death. Proc. Natl. Acad. Sci. USA.

[121] Neary C.L., Pastorino J.G. (2013). Akt inhibition promotes hexokinase 2 redistribution and glucose uptake in cancer cells. J. Cell Physiol..

[122] Gao F., Li M., Liu W.B., Zhou Z.S., Zhang R., Li J.L., Zhou K.C. (2015). Epigallocatechin gallate inhibits human tongue carcinoma cells via HK2-mediated glycolysis. Oncol. Rep..

[123] Hou X., Liu Y., Liu H., Chen X., Liu M., Che H., Guo F., Wang C., Zhang D., Wu J. (2015). PERK silence inhibits glioma cell growth under low glucose stress by blockage of p-AKT and subsequent HK2’s mitochondria translocation. Sci. Rep..

[124] Yoshino H., Enokida H., Itesako T., Kojima S., Kinoshita T., Tatarano S., Chiyomaru T., Nakagawa M., Seki N. (2013). Tumor-suppressive microRNA-143/145 cluster targets hexokinase-2 in renal cell carcinoma. Cancer Sci..

[125] Guo W., Qiu Z., Wang Z., Wang Q., Tan N., Chen T., Chen Z., Huang S., Gu J., Li J. (2015). MiR-199a-5p is negatively associated with malignancies and regulates glycolysis and lactate production by targeting hexokinase 2 in liver cancer. Hepatology.

[126] Qin Y., Cheng C., Lu H., Wang Y. (2016). miR-4458 suppresses glycolysis and lactate production by directly targeting hexokinase2 in colon cancer cells. Biochem. Biophys. Res. Commun..

[127] Jiang S., Zhang L.F., Zhang H.W., Hu S., Lu M.H., Liang S., Li B., Li Y., Li D., Wang E.D. (2012). A novel miR-155/miR-143 cascade controls glycolysis by regulating hexokinase 2 in breast cancer cells. EMBO J..

[128] Gregersen L.H., Jacobsen A., Frankel L.B., Wen J., Krogh A., Lund A.H. (2012). MicroRNA-143 down-regulates Hexokinase 2 in colon cancer cells. BMC Cancer.

[129] Dai W., Wang F., Lu J., Xia Y., He L., Chen K., Li J., Li S., Liu T., Zheng Y. (2015). By reducing hexokinase 2, resveratrol induces apoptosis in HCC cells addicted to aerobic glycolysis and inhibits tumor growth in mice. Oncotarget.

[130] Federzoni E.A., Valk P.J., Torbett B.E., Haferlach T., Löwenberg B., Fey M.F., Tschan M.P. (2012). PU.1 is linking the glycolytic enzyme HK3 in neutrophil differentiation and survival of APL cells. Blood.

[131] Hai-Yan G., Xin-Guo L., Xi C., Jing-Hua W. (2015). Identification of key genes affecting disease free survival time of pediatric acute lymphoblastic leukemia based on bioinformatic analysis. Blood Cells Mol. Dis..

[132] Lu J. (2019). The Warburg metabolism fuels tumor metastasis. Cancer Metastasis Rev..

[133] Jose C., Bellance N., Rossignol R. (2011). Choosing between glycolysis and oxidative phosphorylation: A tumor’s dilemma?. Biochim. Biophys. Acta.

[134] Seiler K., Humbert M., Minder P., Mashimo I., Schläfli A.M., Krauer D., Federzoni E.A., Vu B., Moresco J.J., Yates J.R. (2022). Hexokinase 3 enhances myeloid cell survival via non-glycolytic functions. Cell Death Dis..

[135] Xu W., Liu W.R., Xu Y., Tian X., Anwaier A., Su J.Q., Zhu W.K., Shi G.H., Wei G.M. (2021). Hexokinase 3 dysfunction promotes tumorigenesis and immune escape by upregulating monocyte/macrophage infiltration into the clear cell renal cell carcinoma microenvironment. Int. J. Biol. Sci..

[136] Board M., Colquhoun A., Newsholme E.A. (1995). High Km glucose-phosphorylating (glucokinase) activities in a range of tumor cell lines and inhibition of rates of tumor growth by the specific enzyme inhibitor mannoheptulose. Cancer Res..

[137] Bui N.L.C., Pandey V., Zhu T., Ma L., Basappa L.P.E. (2018). Bad phosphorylation as a target of inhibition in oncology. Cancer Lett..

[138] Danial N.N. (2008). BAD: Undertaker by night, candyman by day. Oncogene.

[139] Jiang P., Du W., Heese K., Wu M. (2006). The Bad guy cooperates with good cop p53: Bad is transcriptionally up-regulated by p53 and forms a Bad/p53 complex at the mitochondria to induce apoptosis. Mol. Cell Biol..

[140] Matschinsky F.M., Magnuson M.A., Zelent D., Jetton T.L., Doliba N., Han Y., Taub R., Grimsby J. (2006). The network of glucokinase-expressing cells in glucose homeostasis and the potential of glucokinase activators for diabetes therapy. Diabetes.

[141] Joseph G., Ramakanth S., Wendy L.C., Nancy-Ellen H., Fred T.B., John W.C., Kevin R.G., Darryl H., Robert K. (2003). Allosteric Activators of Glucokinase: Potential Role in Diabetes Therapy. Science.

[142] Matschinsky F.M. (2009). Assessing the potential of glucokinase activators in diabetes therapy. Nat. Rev. Drug Discov..

[143] Akinobu N and Yasuo T. (2015). Present status of clinical deployment of glucokinase activators. J. Diabetes Investig..

[144] Yoon S.O., Youn-Jung L., Kaapjoo P., Hyun Ho C., Sangjong Y., Hee-Sook J. (2014). Treatment with glucokinase activator, YH-GKA, increases cell proliferation and decreases glucotoxic apoptosis in INS-1 cells. Eur. J. Pharm. Sci..

[145] Porat S., Weinberg-Corem N., Tornovsky-Babaey S., Schyr-Ben-Haroush R., Hija A., Stolovich-Rain M., Dadon D., Granot Z., Ben-Hur V., White P. (2011). Control of pancreatic β cell regeneration by glucose metabolism. Cell Metab..

[146] Kassem S., Bhandari S., Rodríguez-Bada P., Motaghedi R., Heyman M., García-Gimeno M.A., Cobo-Vuilleumier N., Sanz P., Maclaren N.K., Rahier J. (2010). Large islets, beta-cell proliferation, and a glucokinase mutation. N. Engl. J. Med..

[147] Shen Q., Cheng F., Song H., Lu W., Zhao J., An X., Liu M., Chen G., Zhao Z., Zhang J. (2017). Proteome-Scale Investigation of Protein Allosteric Regulation Perturbed by Somatic Mutations in 7,000 Cancer Genomes. Am. J. Hum. Genet..

[148] Těšínský M., Šimčíková D., Heneberg P. (2019). First evidence of changes in enzyme kinetics and stability of glucokinase affected by somatic cancer-associated variations. Biochim. Biophys. Acta Proteins Proteom..

[149] Orci L.A., Sanduzzi-Zamparelli M., Caballol B., Sapena V., Colucci N., Torres F., Bruix J., Reig M., Toso C. (2021). Incidence of hepatocellular carcinoma in patients with nonalcoholic fatty liver disease: A systematic review, meta-analysis, and meta-regression. Clin. Gastroenterol. Hepatol..

[150] Nagaoki Y., Hyogo H., Ando Y., Kosaka Y., Uchikawa S., Nishida Y., Teraoka Y., Morio K., Fujino H. (2021). Increasing incidence of non-HBV- and non-HCV-related hepatocellular carcinoma: Single-institution 20-year study. BMC Gastroenterol..

[151] Li J., Wang J., Chen Y., Yang L., Chen S. (2017). A prognostic 4-gene expression signature for squamous cell lung carcinoma. J. Cell. Physiol..

[152] Yixiang Z., Puyuan X., Junling L. (2016). Treatment of advanced squamous cell lung cancer. Chin. J. Lung Cancer.

[153] Jia H., Wang A., Lian H., Shen Y., Wang Q., Zhou Z., Zhang R., Li K., Liu C. (2020). Identification of novel alternative splicing isoform biomarkers and their association with overall survival in colorectal cancer. BMC Gastroenterol..

[154] Majem M., Juan O., Insa A., Reguart N., Trigo J.M., Carcereny E., García-Campelo R., García Y., Guirado M., Provencio M. (2019). SEOM clinical guidelines for the treatment of non-small cell lung cancer (2018). Clin Transl Oncol..

[155] Anders S., Reyes A., Huber W. (2012). Detecting differential usage of exons from RNA-seq data. Genome Res..

[156] Fuhr L., El-Athman R., Scrima R., Cela O., Carbone A., Knoop H., Li Y., Hoffmann K., Laukkanen M.O., Corcione F. (2018). The Circadian Clock Regulates Metabolic Phenotype Rewiring Via HKDC1 and Modulates Tumor Progression and Drug Response in Colorectal Cancer. EBioMedicine.

[157] Fan L., Huang C., Li J., Gao T., Lin Z., Yao T. (2018). Long non-coding RNA urothelial cancer associated 1 regulates radioresistance via the hexokinase 2/glycolytic pathway in cervical cancer. Int. J. Mol. Med..

[158] Ma Y., Hu M., Zhou L., Ling S., Li Y., Kong B., Huang P. (2019). Long non-coding RNA HOTAIR promotes cancer cell energy metabolism in pancreatic adenocarcinoma by upregulating hexokinase-2. Oncol. Lett..

[159] Nabi K., Le A. (2021). The Intratumoral Heterogeneity of Cancer Metabolism. Adv. Exp. Med. Biol..

[160] Antonio M.J., Zhang C., Le A. (2021). Different Tumor Microenvironments Lead to Different Metabolic Phenotypes. Adv. Exp. Med. Biol..

[161] Evstafieva A.G., Kovaleva I.E., Shoshinova M.S., Budanov A.V., Chumakov P.M. (2018). Implication of KRT16, FAM129A and HKDC1 genes as ATF4 regulated components of the integrated stress response. PLoS ONE.

[162] Lunt S.Y., Vander Heiden M.G. (2011). Aerobic glycolysis: Meeting the metabolic requirements of cell proliferation. Annu. Rev. Cell Dev. Biol..

[163] Schulze A., Harris A.L. (2012). How cancer metabolism is tuned for proliferation and vulnerable to disruption. Nature.

[164] Kamarajugadda S., Stemboroski L., Cai Q., Simpson N.E., Nayak S., Tan M., Lu J. (2012). Glucose oxidation modulates anoikis and tumor metastasis. Mol. Cell Biol..

[165] Ciscato F., Ferrone L., Masgras I., Laquatra C., Rasola A. (2021). Hexokinase 2 in Cancer: A Prima Donna Playing Multiple Characters. Int. J. Mol. Sci..

[166] Xue Y.N., Yu B.B., Li J.L., Guo R., Zhang L.C., Sun L.K., Liu Y.N., Li Y. (2019). Zinc and p53 disrupt mitochondrial binding of HK2 by phosphorylating VDAC1. Exp. Cell Res..

[167] Wang S., Zhuang Y., Xu J., Tong Y., Li X., Dong C. (2023). Advances in the Study of Hexokinase 2 (HK2) Inhibitors. Anticancer Agents Med. Chem..

