# Supplementary material for: Aiding Cancer’s “Sweet Tooth”: Role of Hexokinases in Metabolic Reprogramming
Source: Life (Basel). 2023 Apr 4;13(4):946. doi: 10.3390/life13040946 (PMC10141071; doi:10.3390/life13040946)

Figure Legends:

Supplementary Figure S1: Protein sequence alignment of hexokinase isoforms: Sequence alignment of HK1, HK2, HK3, GCK, and HKDC1 was done using the Uniport website (www.uniport.org), and the percent identity matrix for the five hexokinase isoforms was downloaded.

Supplementary Figure S2: Differential expression hexokinase isoforms: mRNA expression data of HK1, HK2, HK3, GCK, and HKDC1 in various types of cancer in human patients were mined from The Cancer Genome Atlas (TCGA) data set using the GEPIA2 website (http://gepia2.cancer-pku.cn/#index) and used to form a heatmap showing expression of the five HK isoforms in different cancers. Supplementary Table S1: lists all abbreviations for the tumor types in this figure.

Supplementary Table S1: TCGA study abbreviations.

ACC	Adrenocortical carcinoma
BLCA	Bladder Urothelial Carcinoma
LGG	Brain Lower Grade Glioma
BRCA	Breast invasive carcinoma
CESC	Cervical squamous cell carcinoma and endocervical adenocarcinoma
CHOL	Cholangiocarcinoma
LCML	Chronic Myelogenous Leukemia
COAD	Colon adenocarcinoma
CNTL	Controls
ESCA	Esophageal carcinoma
FPPP	FFPE Pilot Phase II
GBM	Glioblastoma multiforme
HNSC	Head and Neck squamous cell carcinoma
KICH	Kidney Chromophobe
KIRC	Kidney renal clear cell carcinoma
KIRP	Kidney renal papillary cell carcinoma
LIHC	Liver hepatocellular carcinoma
LUAD	Lung adenocarcinoma
LUSC	Lung squamous cell carcinoma
DLBC	Lymphoid Neoplasm Diffuse Large B-cell Lymphoma
MESO	Mesothelioma
MISC	Miscellaneous
OV	Ovarian serous cystadenocarcinoma
PAAD	Pancreatic adenocarcinoma
PCPG	Pheochromocytoma and Paraganglioma
PRAD	Prostate adenocarcinoma
READ	Rectum adenocarcinoma
SARC	Sarcoma
SKCM	Skin Cutaneous Melanoma
STAD	Stomach adenocarcinoma

Supplementary Figure S1

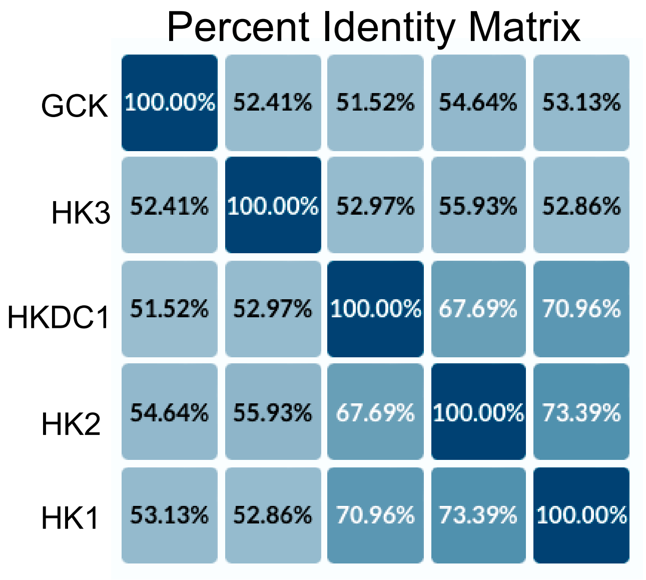

Supplementary Figure S2

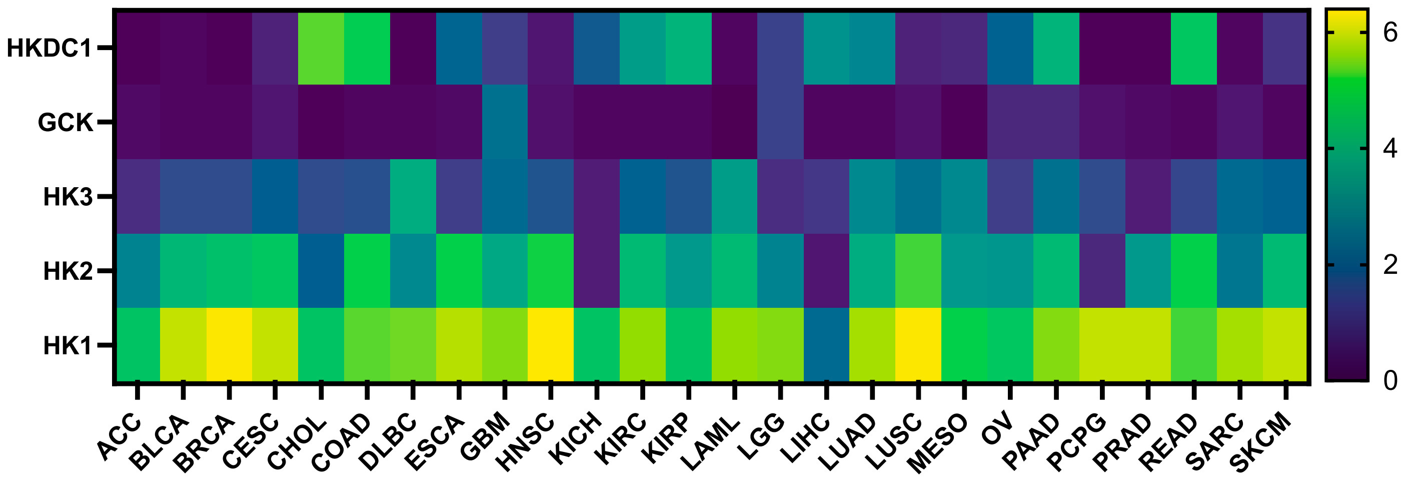

Supplement: Supplementary file 1 [file life-13-00946-s001.zip › life-2269875-supplementary.pdf]
